# Influenza

**Published:** 1898-02-12

**Authors:** 


### INFLUENZA.

The wide prevalence of influenza at the present time renders it of more than usual importance that we should be prepared with answers to two questions—(1) Should isolation be attempted, and (2) does the occurrence of one attack in any way protect a patient from being attacked again ?

As to the first it is clearly a question of practicability, and we have very little hesitation in saying that any such isolation as would be likely to interfere in any useful degree with the dissemination of the disease is quite impracticable. Even if we look back to the time nine years ago when the disease came upon us as a new thing—new at least to the present generation—and when, if ever, it ought to have been possible to restrain its progress, we find that its onset in the towns attacked was so sudden and widespread that it seemed to many people quite out of the question to attribute it to an infection passing from case to case; and although on looking back upon these outbreaks, and tracing out their history, it was often possible to discover the manner in which the disease had been introduced, it became abundantly clear that any rules or regulations sufficiently stringent to have caught the individual who did the mischief would have interfered in an unwarrantable manner with scores of others who were perfectly harmless. The fact is that those who are laid up by influenza form but a portion of those attacked, and that the main distribution of the infection takes place by fomites and by cases that walk about and endeavour to do their work. There are, however, two other facts that have to be borne in mind in regard to the possibility of preventing the spread of influenza by isolation; one that, as Goodhart says, like measles, it infects actively so early that it is at work for this purpose long before it can be recognised; and the other, that it is a disease which presents so many types and presents such a variety of symptoms, often far from characteristic, that many cases occur in which, however strongly we may believe influenza to be present, such certainty as would justify us shutting a man up is entirely wanting.

In regard to the question of the protective influence of one attack against another, it seems quite certain that, if it occurs at all, it is of very short duration. Dr. Goodhart says that not only does an attack not render the patient immune, but that certain persons seem to be attacked preferentially, as it were, again and again. Dr. Parsons, no doubt, shows that a severe epidemic of influenza appears to confer upon a locality a certain degree of protection against a repetition; but then, in saying this, he is dealing with death-rates, which are a somewhat different matter. The number of people who are liable to be killed by influenza is comparatively small, and a first epidemic makes such havoc in the ranks of the old and feeble "susceptibles" that it takes some years to replace them.

Writing on this question Dr. Turney says that during the first week of the epidemic of 1891 as many as 13·4

per cent. of his out-patients suffering from influenza had previously had the disease, and that as weeks went by the proportion of cases in which a previous attack could be traced gradually diminished, so that it would appear as if not only did a previous attack not protect against a repetition, but as if there were certain persons so susceptible as to be picked out at once by the infection for a second attack. Certain people are undoubtedly far less susceptible to the poison of influenza than others, but their good fortune is due to a something in their constitution or their surroundings, not to any protection derived from a previous attack.

